# Effect of Social Support in Pain Sensitivity in Children with Cerebral Palsy and Typically Developing Children

**DOI:** 10.3390/ijerph18094661

**Published:** 2021-04-27

**Authors:** Inmaculada Riquelme, Isabel Escobio-Prieto, Ángel Oliva-Pascual-Vaca, Alberto Marcos Heredia-Rizo, Pedro Montoya

**Affiliations:** 1 Institute of Health Sciences Research (IUNICS-IdISBa), University of Balearic Islands, Carretera de Valldemossa km 7.5, 07122 Palma de Mallorca, Spain; pedro.montoya@uib.es; 2 Department of Nursing and Physiotherapy, University of the Balearic Islands, 07122 Palma de Mallorca, Spain; 3 Departamento de Fisioterapia, Facultad de Enfermería, Fisioterapia y Podología, Universidad de Sevilla, 41009 Sevilla, Spain; iescobio@us.es (I.E.-P.); angeloliva@us.es (Á.O.-P.-V.); amheredia@us.es (A.M.H.-R.)

**Keywords:** cerebral palsy, social support, pain perception, healthy peers

## Abstract

Pain and abnormal somatosensory processing are important associated conditions in children and adolescents with cerebral palsy (CP). Perceived social support is highly relevant for pain perception and coping. Aim: The aim of the present study was to assess the influence of social support on pain sensitivity in children and adolescents with cerebral palsy and healthy peers. Design: Cross-sectional study. Methods: Pressure pain thresholds were assessed in 42 children and adolescents with CP and 190 healthy peers during three different conditions: alone, with their mother and with a stranger. Results: Children with CP reported lower pain sensitivity when they were with their mother than being alone or with a stranger, whereas healthy peers did not experience different pain sensitivity related to the social condition. Sex or clinical characteristics did not affect the relationship between pain perception and social support. Conclusion: The present study shows how children with CP are highly affected by social and contextual influences for regulating pain sensitivity. Solicitous parental support may enhance pain perception in children with CP. Further research on the topic is warranted in order to attain well-founded conclusions for clinical practice.

## 1. Introduction

Pain is a significant problem in children and adolescents with cerebral palsy (CP). More than 50% of children with CP suffer pain from moderate to severe intensity and at multiple body locations [1,2]. Pain negatively affects quality of life [2,3] and participation [2,4]. Furthermore, individuals with CP have altered somatosensory processing and pain sensitivity [5], requiring the development of specific coping strategies to manage pain [6].

Although some coping strategies, such as behavioral distraction, can help to reduce pain, maladaptive coping may be a risk factor for poor psychosocial and functional outcomes [7]. Perceived social support and solicitous responding have been considered among the most relevant factors contributing to the maintenance of pain in people with physical disabilities [8]. Children and adolescents with CP perceived that pain limited their social support [9], and ambulatory children with CP suffering pain made important efforts to achieve social acceptance [10]. Nevertheless, seeking social support has also been interpreted as a maladaptive coping strategy, since it may signal a lack of ability to deal with pain [10,11,12]. In this sense, it has been shown that social support was a predictor of disability in adults with CP [13] and children with developmental disabilities [11].

The presence of significant others seems to influence pain perception and the effectiveness of social support. Thus, more pain behaviors and increased sensitivity to noxious stimuli, such as thermal or pressure pain thresholds, have been reported during high-empathic conditions, such as the presence of a familiar or partner, than in low- or non-empathic conditions, such as being alone or in the presence of a stranger [14,15,16,17]. Parents are powerful regulators of pain stress in children [18]. In this line, maternal presence (compared to maternal absence) increased heat pain thresholds in school-aged children, although this presence did not modulate pain habituation in children with a history of neonatal hospitalization [19]. Parents’ reassurance and protectiveness in painful situations has been also associated with increased distress and pain; thus, some studies have shown that children were more likely to exhibit pain behaviors after maternal solicitousness [19,20,21,22]. Nevertheless, there is no study addressing the question of how the presence of significant others could regulate pain sensitivity in children with CP. In the present study, we aimed at measuring pressure pain thresholds in children and adolescents with CP and healthy peers during three different social conditions: alone, in the presence of their mother, and in the presence of a stranger.

## 2. Materials and Methods

### 2.1. Study Design

This is a cross-sectional study. The study period was from July 2019 to September 2020. The research protocol was approved by the Ethics Committee of the Regional Government of the Principado de Asturias (ref. 220/19). This study was carried out in accordance with the Declaration of Helsinki (2013). Before the inclusion, all parents signed an informed consent and the participants also gave their oral approval to participate.

### 2.2. Participants

Children and adolescents with cerebral palsy (CP) and healthy peers were recruited from educational and leisure centers established in the Principado de Asturias (Spain) between 2019 and 2020. A group of 50 potential participants with CP was initially identified by their own physicians. These children and healthy peers of the same age were invited to participate using an informational letter explaining the details of the research study. This letter was provided to the families during the children’s attendance to the educational/leisure activities, in person or through the school diary. Inclusion criteria were: (1) age between 4 and 16 years old and (2) cognitive level that allows understanding the instructions and procedure (i.e., answer when they felt pain upon stimulation). Augmentative communication devices and information from caregivers were used as needed to facilitate data collection in participants with communication difficulties.

### 2.3. Procedure and Measures

**Figure 1:**
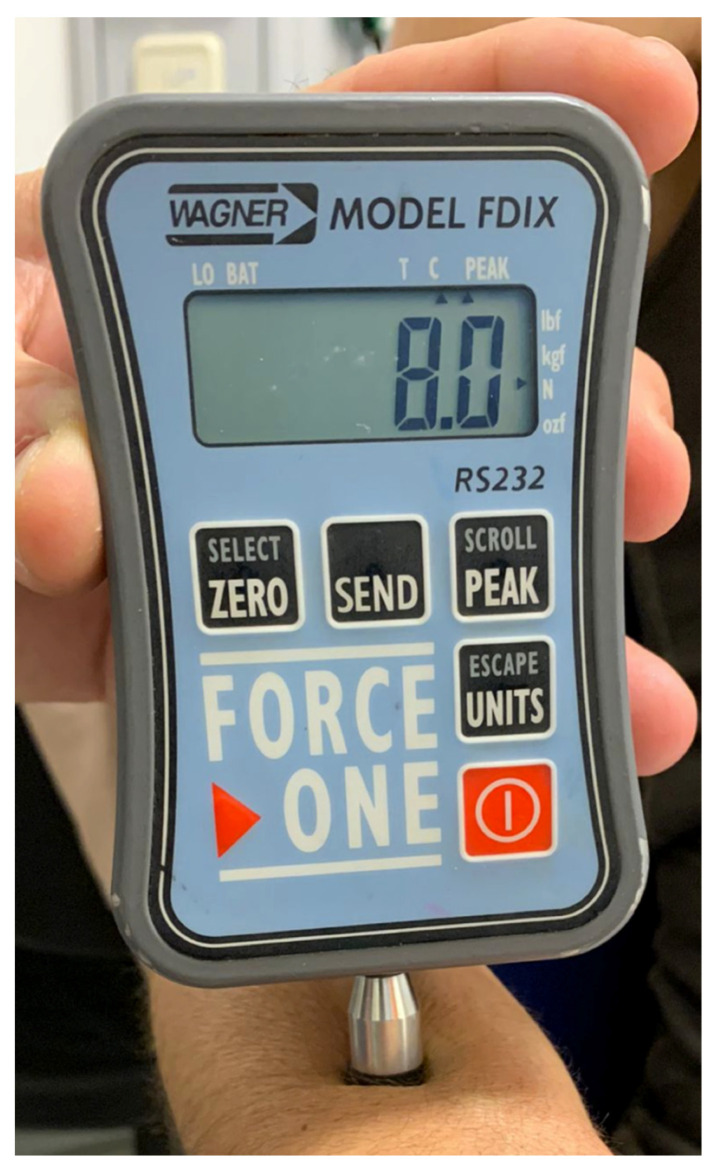
Assessment of pressure pain thresholds.

Pressure pain thresholds were determined bilaterally on the dorsal middle part of forearms (Figure 1), as this body location has not been related to pain in individuals with cerebral palsy and thus, can better expose central sensitivity. Pressure pain thresholds (expressed in Newtons) were measured with a digital dynamometer using a flat rubber tip (surface of the tip: 1 cm^2^). Children were asked to say “yes” or to use their usual gesture for saying “yes” (e.g., turning the head) when the pressure became painful (pressure pain threshold). Pressure was released when the pain threshold or the maximally exerted pressure of the dynamometer was reached. Three stimuli were applied at each body location and the average of all measurements was calculated as the pressure pain threshold for each body location. The average of both body locations (left and right forearm) was considered as the pressure pain threshold. To avoid anxiety, at the start of the experimental session, children were familiarized with the assessment procedure by using several non-painful stimuli in the forearms. All children correctly understood and pursued the procedure and no participant expressed distress during its execution. The reliability of this procedure for assessing pressure pain sensitivity [23] and the reliability of the capacity to express pain by children with cognitive deficits has been shown in previous studies [24,25].

The same researcher examined pressure pain thresholds during three experimental conditions: (1) participants were seated alone, (2) participants were seated with their mother and (3) participants were seated with an unknown adult person (stranger). The accompanying person (mother and stranger) was asked to maintain eye contact with the subject and to remain in silence. Testing order of stimuli and conditions was randomized. The assessment was performed individually in a quiet, isolated room at the child’s school/leisure center. The total duration of the procedure was fifteen minutes.

### 2.4. Statistical Analysis

For comparing children with CP with healthy peers, two-way ANOVAs including the between-subject factor group (children with CP vs. healthy peers) and the within-subject factors condition (alone vs. mother vs. stranger) were performed. Additional analyses of covariance (ANCOVAs) were performed for controlling for the effects of sex (boys vs. girls). Pearson and Spearman correlations were performed for exploring associations among pain in the different conditions and clinical data in children with CP. ANOVA results were adjusted by using Bonferroni corrections for post-hoc comparisons. Significance levels were set at *p* < 0.05.

## 3. Results

### 3.1. Description of the Sample

A total of forty-two children and adolescents with CP (16 females; mean age = 9.19 (SD = 2.95), range 4–16 years), and 190 healthy peers (98 females; mean age = 10.74 (SD = 3.04), range 5–16 years) were recruited and decided to participate in the study. Children or their parents reported children’s age and sex. The type of cerebral palsy, gross and manipulative motor function, cognitive level and type of educational setting were obtained from medical records. None of the participants had pain at the moment of the assessment. Table 1 displays clinical characteristics of participants with CP. As regular and special schools were included in the recruitment, our sample encompassed a broad range of motor impairment. The data presented in this study are available on request from the corresponding author.

### 3.2. Analysis of Outcome Measures

**Figure 2:**
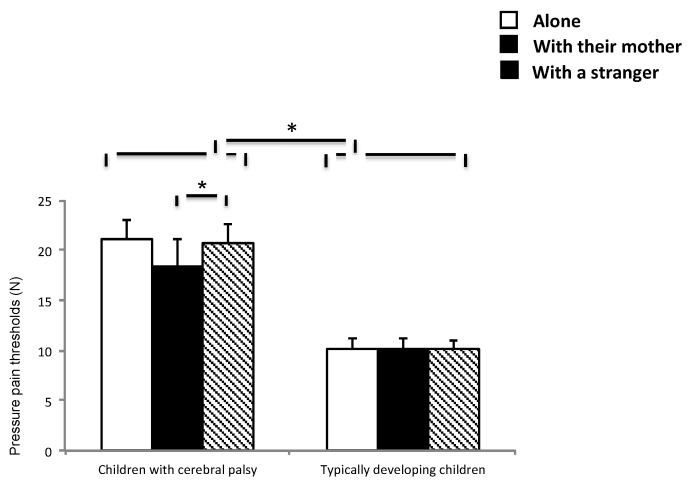
Pressure pain thresholds for each group (children with CP vs. healthy children) at the three social support situations (alone, with their mother, or with a stranger). Results are displayed as mean ± TE. ANOVA: * *p* < 0.05.

Figure 2 displays the descriptive data of pain thresholds for both groups in the three conditions, and pairwise statistical results are displayed in Table 2 and Table 3. A significant interaction effect of group X condition (F(2202) = 3.32, *p* = 0.045) revealed higher pain thresholds (lower pain sensitivity) in children with CP than in healthy peers (all *p* < 0.015); in addition, children with CP showed lower pain thresholds if they were with their mother than when they were with a stranger (*p* = 0.036), whereas no significant differences among conditions were found in healthy peers. A significant main effect for group (F (1208) = 6.81, *p* = 0.01) showed higher thresholds in children with CP than in healthy peers. A non-significant trend in the main effect for condition (F (2207) = 9.97, *p* = 0.061) showed lower pain thresholds when children were with their mother than when they were with a stranger. 

Analysis of covariance (ANCOVAs) controlling for the effects of sex yielded the same significant effects (all F > 3.08, all *p* < 0.048).

No significant correlations were found among pain in the different conditions and clinical characteristics in children with CP.

## 4. Discussion

The aim of the present study was to explore how children with cerebral palsy and healthy peers regulate pain sensitivity in different conditions of social support: alone, in the presence of their mother, or in the presence of a stranger. Children with CP reported lower pain sensitivity when they were with their mother than being with a stranger, whereas no differences on pain sensitivity were found among the three social conditions in healthy peers.

The present study revealed that the presence of a significant other (mother) increased pain sensitivity in children with CP. Although children with CP have multiple pain experiences and increased pain sensitivity from an early age [24], they seem to highly rely on social support and on a low use of other active strategies for coping with this pain [6], in contrast to healthy peers who use a greater range of adaptative pain-coping strategies. Previous research has shown that perceived social support may regulate physiological stress responses and pain ratings in a healthy population [16,17,28]. Moreover, some studies in healthy adults have reported that the presence of a partner or familiar would lead to an increment of pain sensitivity [19,20] and some authors considered seeking of social support and parental solicitousness as a maladaptive coping strategy in children with chronic pain and individuals with developmental disabilities [12,13,22,29]. This hypothesis is confirmed by our findings, as the mother’s presence enhanced pain perception in children with cerebral palsy, instead of acting as a regulator of pain stress. This fact has implications for the intervention in children with CP, which must promote the use of active cognitive pain-coping strategies, such as problem solving, cognitive self-instruction, creation of mind-body disassociation and task perseverance.

Although our results were not affected by sex or the clinical condition, children with CP reported lower pain sensitivity than healthy peers. These findings are surprising, as all previous studies had shown that individuals with CP had increased pain sensitivity and altered somatosensory processing [5]. The environment where data were collected, that in case of children with CP was the school, where also therapeutic interventions are performed, could have caused this discrepancy. As children with CP perceive many interventions as a source of pain [30] and apprehension of the therapy sessions can further increase pain perception [30,31], the execution of a new assessment procedure could have induced pain anxiety in our sample of children with CP. Unpublished data from our lab showed that children with CP used pain-coping strategies more frequently than their age-related peers, due to the frequent pain situations suffered from early ages (Riquelme et al., submitted). Thus, paradoxically, our assessment might have generated pain-coping responses in children with CP, similar to those used for the painful interventions provided in that environment. This fact highlights the influence of contextual events in children with CP, reinforcing the notion of social dependency and reduced ability for self-efficacy in pain coping.

The main limitation of the present study is that we did not have information about personality traits, such as the personal attachment style or empathy, which have shown to influence pain regulation in healthy adults [15,32]. Our social supporters remained in silence, as we tried to reduce the effects of distraction or solicitous attitudes in the assessments [21]; however, verbal support has been proven as being more efficient than only the mere presence of an individual in reducing pain sensitivity [28]; thus, pain regulation may be different in ecological social contexts. Pain perception may also be modulated by a wide range of factors, such as age, comorbidities, etc., which influence could not be analyzed in depth due to the small number of our sample with CP. While sex was controlled in the statistical analysis, our sample was formed by significantly more males than females and we cannot discard sex-related potential bias. Although all children were able to communicate pain verbally or by means of augmentative communication, no specific scale for assessing communication was used.

## 5. Conclusions

In the present study, we can conclude that children with CP seem dependent on social influence for regulating pain sensitivity. Solicitous parental support may enhance pain perception in children with CP, instead of acting as a regulator of pain stress. Further research on the topic is warranted in order to attain well-founded conclusions for clinical practice.

## Figures and Tables

**Table 1:** Demographic and clinical characteristics of the children and adolescents with cerebral palsy.

Clinical Variable	N
Gender	
Male	26
Female	16
Type of cerebral palsy	
Bilateral spastic	28
Diskinetic	14
Other (unilateral spastic, ataxic, mixed)	0
Less affected side	
Right	34
Left	8
Motor impairment (GMFCS)	
Level I	6
Level II	8
Level III	8
Level IV	4
Level V	16
Manual impairment (MACS)	
Level I	8
Level II	4
Level III	12
Level IV	2
Level V	16
Cognitive impairment	
None	26
Mild	2
Moderate	6
Severe	8
Type of schooling	
Regular school	24
Special school	18

GMFCS = Gross Motor Function Classification System [26], describes the gross motor function according to 5 levels: 1 = walks without limitations, 5 = transported in a manual wheelchair. MACS = Manual Ability Classification System [27], describes the use of hands in daily activities according to 5 levels: 1 = handles objects easily and successfully, 5 = does not handle objects and has severely limited ability to perform even simple actions.

**Table 2:** Statistical pairwise comparisons of pressure pain thresholds for each group (children with CP vs. healthy children) at the three social support situations (alone, with their mother, with a stranger).

	Mean Difference	Standard Error	Significance Level	95% Confidence Interval for Difference
Alone	7.25	2.91	0.013	1.52/12.97
With their mother	8.37	2.96	0.005	2.54/14.20
With a stranger	6.97	2.85	0.015	1.35/12.60

**Table 3:** Statistical pairwise comparisons of pressure pain thresholds for each social support situations (alone vs. with their mother vs. with a stranger) at each group (children with CP, healthy children).

	Mean Difference	Standard Error	Significance Level	95% Confidence Interval for Difference
**Healthy children**				
Alone vs. with mother	0.03	0.21	1.0	−0.49/0.54
Alone vs. with stranger	−0.01	0.15	1.0	−0.37/0.35
Mother vs. stranger	−0.04	0.18	1.0	−0.47/0.39
**Children with cerebral palsy**				
Alone vs. with mother	−1.09	0.64	0.27	−2.63/0.45
Alone vs. with stranger	0.27	0.44	1.0	−0.80/1.33
Mother vs. stranger	1.36	0.54	0.036	0.063/2.65

## Data Availability

The data presented in this study are available on request from the corresponding author.
